# Prevalence of Posttranscatheter Aortic Valve Implantation Vascular Complications in Real Life

**DOI:** 10.1155/2021/5563486

**Published:** 2021-10-12

**Authors:** Anthony Matta, Ronan Canitrot, Vanessa Nader, Frederic Bouisset, Thibault Lhermusier, Francisco Campelo-Parada, Etienne Grunenwald, Bertrand Marcheix, Meyer Elbaz, Didier Carrie, Jerome Roncalli

**Affiliations:** ^1^Department of Cardiology, Institute CARDIOMET, University Hospital of Toulouse, Toulouse, France; ^2^Faculty of Medicine, Holy Spirit University of Kaslik, Kaslik, Jounieh, Lebanon; ^3^Faculty of Pharmacy, Lebanese University, Beirut, Lebanon; ^4^Department of Cardiac Surgery, Institute CARDIOMET, University Hospital of Toulouse, Toulouse, France

## Abstract

**Background:**

Vascular complications (VCs) are commonly observed after transfemoral transcatheter aortic valve implantation (TAVI) procedures. Closure devices for the access site were developed to reduce their incidence. We aim to evaluate the prevalence, predictors, and outcomes of the occurrence of post-TAVI VCs.

**Materials and Methods:**

A retrospective study was conducted on 1336 consecutive patients who underwent TAVI at the University Hospital of Toulouse, France, between January 2016 and March 2020. All included procedures were performed through the common femoral artery, and ProGlide^®^ was the used closure device. The studied population was divided into two groups depending on the occurrence of VCs defined according to Valve Academic Research Consortium-2 criteria.

**Results:**

The mean age of the studied population was 84.4 ± 6.9, and 48% were male. 90% of TAVI interventions were performed through the right femoral artery. The prevalence of VCs was 18.8%, and 3.7% were major. Prolonged procedure duration was an independent predictor of VCs. Using the right access site and smaller introducer size (14 Fr) were preventive factors. No significant difference in mortality rate was detected between the two groups.

**Conclusion:**

This study showed a low prevalence for post-TAVI VCs, especially for the major type. An increase in bleeding events and prolonged cardiac care unit stay were the common adverse outcomes.

## 1. Introduction

Degenerative aortic stenosis is the most common valvular heart disease affecting more than 2% of the population aged above 65 years [1, 2]. Surgical valve replacement is the traditional recommended therapeutic approach. Recently, transcatheter aortic valve implantation (TAVI) has revolutionized the management of severe aortic stenosis, and its indication was extended to low-surgical-risk patients [3]. TAVI becomes the intervention of choice in high-operative-risk patients and an alternative option for those at low and intermediate risk [4–6].

Over eighteen years of experience, the efficacy and safety of the TAVI procedure have been established. TAVI is gaining ground in the management of aortic stenosis for several factors such as minimally invasive intervention performed under local anesthesia and the new technical advances. Instead, a higher rate of vascular complications is observed after TAVI compared to surgical valve replacement [7]. The common femoral artery is the most often used artery, and different local closure system devices were developed to reduce the occurrence of access site complications. The use of the ProGlide^®^ closure device prevailed during transfemoral TAVI.

Herein, we evaluate the prevalence of vascular complications after transfemoral TAVI using ProGlide^®^ for access site closure and their impact on periprocedural outcomes.

## 2. Materials and Methods

### 2.1. Study Design and Population

**Figure 1:**
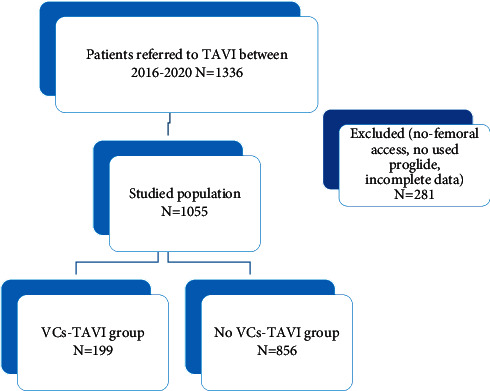
Study flowchart.

An observational retrospective study was conducted on 1336 consecutive patients referred for TAVI at the structural and interventional cardiology department at the University Hospital of Toulouse, France, between January 2016 and March 2020. Patients with nontransfemoral TAVI (transaortic, transcarotid, transapical, and subclavian) or with incomplete data were excluded from the study. The included TAVI procedures during the fixed period of time were performed through the right or left common femoral artery by the same senior operators. The crossover technique for vascular access was systematically performed before device implantation. Vascular sheaths (7 Fr) were placed in both femoral arteries, preclosing the selected femoral artery for valve delivery via two ProGlide^®^ prior to upsizing the sheath to 14–20 Fr. After that, intravenous 5000 IU of heparin was given. A standard right ventricular stimulation (temporary pacing) was conducted while implanting the valve. Following successful valve implantation, ProGlide^®^ was the used ipsilateral femoral access closure device in all included patients, and FemoSeal^®^ was used for the contralateral access site after protamine sulfate administration. It is noteworthy that an angiographic control of the femoral access site is consistently conducted to assess for vascular complications before the closure process. All TAVI procedures were conducted in the presence of an interventional cardiologist, cardiovascular surgeon, and cardiac anesthesiologist. Depending on the occurrence of vascular complications (VCs), the studied population was divided into 2 groups: those with post-TAVI vascular complications versus others (Figure 1). Vascular complications were defined according to Valve Academic Research Consortium-2 criteria [1].

### 2.2. Data Collection and End Points

Data concerning baseline characteristics (age and sex), cardiovascular risk factors (diabetes mellitus, systemic hypertension, smoking, dyslipidemia, and BMI), medical treatment (aspirin, P2Y12 inhibitors, and oral anticoagulant), previous medical history (prior MI, PCI, CABG, stroke, carotid, and peripheral artery disease), concomitant comorbidities (chronic respiratory disease, renal replacement therapy, and atrial fibrillation), and previous valvuloplasty and TAVI procedure (indication, femoral access site, introducer sheath size, valve sizes, valve types, and procedure duration) were collected. The study has been conducted according to the principles outlined in the Declaration of Helsinki. Post-TAVI adverse clinical outcomes were defined as death from any cause, bleeding (minor, major, or life-threatening), vascular complications (minor or major), stroke, pacemaker implantation, and cardiac care unit admission. We primarily aim to determine the incidence of post-TAVI vascular complications, the associated predictors, and post-TAVI adverse clinical outcomes listed above.

### 2.3. Statistical Analysis

Categorical variables were summarized as number and percentage and continuous variables as mean ± standard deviations. Continuous variables were compared with the use of the *t*-test, as appropriate, and categorical variables with the use of *χ*^2^ or Fisher's exact test, as appropriate. A stepwise logistic regression analysis including all variables with *p* value <0.2 in the univariate analysis comparing the post-TAVI vascular complications' group to no post-TAVI vascular complications' group was conducted to assess predictors and adverse clinical outcomes significantly associated with the occurrence of post-TAVI vascular complications. A two-sided *p* value <0.05 was considered as statistical significance. All statistical analyses were carried out by using SPSS version 20.

## 3. Results

Out of 1336 consecutive patients who underwent TAVI, 1055 were eligible for inclusion in the study, and 281 were excluded. Baseline and demographic characteristics of the studied population are shown in Table 1. The mean age was 84.4, and 48% of patients were male. The population was at a higher surgical risk with a predicted mortality of 6 ± 5.5 by STS-PROM and of 14.2 ± 9.9 by EuroSCORE1. Over half of the studied population were classified at NYHA II. The prevalence of population distribution by cardiovascular risk factors was 69.5% for systemic hypertension, 27.4% for diabetes mellitus, 42.7% for dyslipidemia, and 2.1% for smoking. Concerning previous medical history, the prevalence of myocardial infarction, stroke, peripheral artery disease, carotid artery disease, atrial fibrillation, and chronic respiratory disease was 8.2%, 10.4%, 7.4%, 3.5%, 37.8%, and 17.8%, respectively. Prior percutaneous coronary intervention, coronary artery bypass graft, and valvuloplasty were, respectively, observed in 19.8%, 6%, and 11.2% of the whole population. Moreover, 19.1% of the studied population did not receive any antithrombotic treatment, and 26.4% received single antiplatelet therapy and 19.7% dual antiplatelet therapy. Overall, 24.3% were on an anticoagulant therapy alone and 10.5% in combination with antiplatelet therapy.

Most TAVI procedures were performed for severe aortic stenosis (93.5%) using the right femoral access site in 90% of cases. The introducer sheath size varies from 14 Fr (59.9%) to 20 Fr (0.9%). The implanted valves were Edwards SAPIEN (51%), CoreValve Evolut (44.2%), and ACURATE (4.8%). Implanted valves' diameters range from less than 25 mm (26.3%) to more than 30 mm (8.6%). Most of TAVI procedures' duration was between 60 and 120 min. The characteristics of TAVI procedures are shown in Table 2.

**Figure 2:**
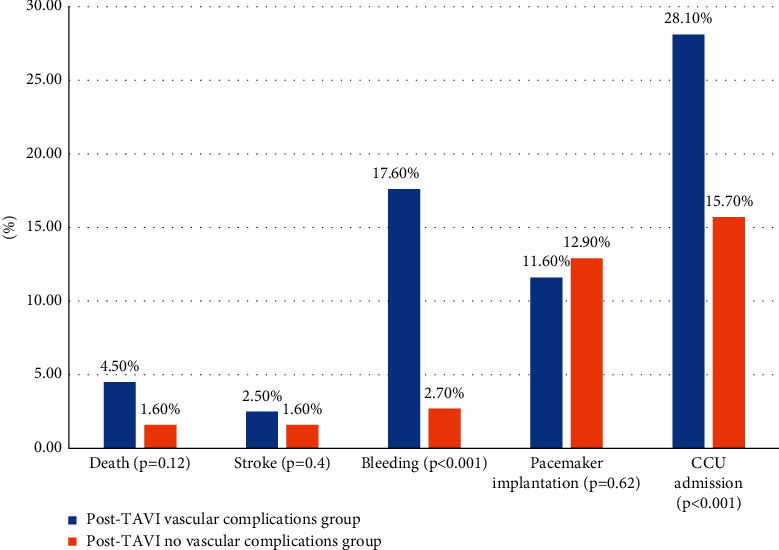
Univariate analysis comparing the prevalence of post-TAVI complications between the two groups.

Post-TAVI vascular complications (VCs) were observed in 18.8% of the entire population, and 3.7% were major (Table 3). The vascular complications have been treated by simple external compression, balloon inflation for residual stenosis or mild leakage, covered stent implantation (15.6%), or surgical approach (1.5%). We note that only three procedures were converted from percutaneous to open-heart surgery for severe vascular complications (aortic rupture). Then, the studied population was divided into 2 groups: post-TAVI VC group (*N* = 199) and post-TAVI with no-VC group (*N* = 856). Except for pacemaker implantation, post-TAVI adverse clinical outcomes including death, stroke, bleeding, and cardiac care unit admission were more common in the post-TAVI vascular complications' group (Figure 2). Univariate analyses have shown significant differences at the 0.2 level between the two groups in terms of distribution of systemic hypertension, smoking, NYHA class, previous valvuloplasty, carotid artery disease, BMI, antithrombotic regimen, right access site, introducer sheath size, valve types, procedure duration, death, bleeding, and cardiac care unit stay (Tables 1 and 2 and Figure 2). After adjusting for confounding variables listed above, the multivariate logistic regression showed that longer procedure duration (more than 120 min) is an independent predictor for VCs (OR = 1.8; 95% CI = [1.2; 2.8]) (Table 4). Also, negative associations between VCs and right access site (OR = 0.6; 95% CI = [0.37; 0.99]) and smaller introducer sheath size 14 Fr (OR = 0.63; 95% CI = [0.45; 0.89]) have been shown. Post-TAVI vascular complications' group was more predisposed to bleeding (OR = 2.57; 95% CI = [1.8; 3.6]) and prolonged cardiac care unit stay (OR = 2; 95% CI = [1.4; 3]).

## 4. Discussion

This study showed an overall prevalence of post-TAVI VCs at 18.8% while using the ProGlide^®^ device for the closure of the femoral access site. Compared to the old manual compression method, closure device systems are less painful and provide faster hemostasis, earlier mobilization, and discharge [8]. Indeed, Perclose ProGlide^®^ device is used increasingly worldwide during TAVI procedures and endovascular aneurysm repair interventions [8]. A current published study showed an incidence of post-TAVI vascular complications at 21% [9], and similar rates were found in previously published studies [10–13]. The prevalence of major vascular complications was 3.7%, while most reported values in the literature range between 1.9% and 30.7% [9, 14–18]. The lowest prevalence revealed by our study may be related to the largest sample size, new valve generations, older age of the studied population requiring delicate and careful manipulations, operators' experience, and homogeneity of the closure device system (exclusively Perclose ProGlide^®^). A similar rate at 3.4% of post-TAVI VCs was reported in a study comparing the use of Perclose ProGlide between patients undergoing TAVI and endovascular aneurysm repair [8]. The higher prevalence of minor VCs compared to the major VCs is due to the VARC-2 definition classifying all postprocedural access site hematomas into minor VCs.

Smaller sheaths (14 Fr) were significantly associated with a lower rate of VCs in accordance to what was previously reported [9, 19]. Prior studies identified sheaths above 19 Fr as an independent predictor for VCs [16, 20, 21]. Usually, the choice of sheaths is influenced by the valve type and size. In fact, two-thirds of used valves through smaller sheaths (14 Fr) were balloon-expandable (63.4%), while self-expandable valves (67.5%) were often used in larger sheaths. Taking access through the right common femoral artery is a preventive factor compared to the left side on the occurrence of VCs. This point comparing both access sites is described for the first time in the literature [8–18, 22]. Technical parameters such as the operator to patient position and routine behavior may explain this significant difference in favor for the right access site. Also, it is worthy to mention that the right access site is used by default in real life. Prolonged procedure duration (>120 min) is an independent predictor for the occurrence of VCs, and systemic hypertension showed a strong trend toward increased risk. We believe that our study is the largest one to report on the prevalence and clinical relevance of vascular complications after transfemoral TAVI procedures.

Lastly, worse clinical outcomes were attributed to the occurrence of VCs after TAVI [19]. An increased length of hospital stay and reduced quality of life were reported [14, 16, 18, 20]. The impact of VCs on early mortality is controversial: numerous studies reported an increase in mortality rate [18, 20, 23], whereas no statistical difference was observed in others [24]. Herein, we revealed a significant association with bleeding events and prolonged stay in the cardiac care unit (>24 hours) with no effect on overall mortality rate.

### 4.1. Limitations

The limitations of the study were the retrospective monocentric nature. Despite the largest sample size, the low prevalence of post-TAVI vascular complications may limit the ability to detect all independent factors. The lack of performing a systemic echo Doppler for the access site after transfemoral TAVI may result in a subjective and misestimation of minor vascular complications. Lastly, CT-scan data were not collected knowing that, except for artery calcification, no predictor factor was previously identified in the literature.

## 5. Conclusion

Vascular complications after transfemoral TAVI procedures constitute the main safety limitations. New valve generations, experienced operators, and closure system devices lead to a dramatic decrease in their incidence. We believe that our study provides an up-to-date on the prevalence, predictors, and impact of post-TAVI VCs in real life. To conclude, using an appropriate sheath size in accordance with the valve type, shortening procedure time, and giving preference for the right access site are daily important parameters. Increased rate of bleeding events and prolonged cardiac care unit stay are the major observed adverse outcomes.

## Figures and Tables

**Table 1:** Baseline characteristics of the studied population.

	Whole population (*N* = 1055)	Post-TAVI vascular complication group (*N* = 199)	Post-TAVI without vascular complication group (*N* = 856)	*p* value
Age (mean ± SD)	84.4 ± 6.9	84.8 ± 7.3	84.3 ± 6.9	0.31

Sex (*N*, %)				
Male	506 (48)	97 (48.7)	409 (47.8)	0.8

CVRF (*N*, %)				
Systemic hypertension	733 (69.5)	150 (75.4)	538 (68.3)	0.04
Diabetes mellitus	289 (27.4)	58 (29.1)	231 (27)	0.53
Dyslipidemia	450 (42.7)	91 (45.7)	359 (41.9)	0.33
Smoker	22 (2.1)	7 (3.5)	15 (1.8)	0.11

NYHA class (*N*, %)				0.08
I	16 (1.5)	3 (1.5)	13 (1.5)	
II	574 (54.4)	124 (62.3)	450 (52.6)	
III	415 (39.3)	63 (31.7)	352 (41.1)	
IV	50 (4.7)	9 (4.5)	41 (4.8)	

Previous medical history (*N*, %)				
Myocardial infarction	86 (8.2)	15 (7.5)	71 (8.3)	0.72
PCI	209 (19.8)	38 (19.1)	171 (20)	0.77
Valvuloplasty	118 (11.2)	29 (14.6)	89 (10.4)	0.09
CABG	63 (6)	11 (5.5)	52 (6.1)	0.76
Stroke	110 (10.4)	20 (10.1)	90 (10.5)	0.84
Peripheral artery disease	78 (7.4)	11 (5.5)	67 (7.8)	0.26
Carotid artery disease	37 (3.5)	10 (5)	27 (3.2)	0.19
Atrial fibrillation	399 (37.8)	71 (35.7)	328 (38.3)	0.48
Chronic respiratory disease	188 (17.8)	30 (15.1)	158 (18.5)	0.26

Antithrombotic therapy (*N*, %)				
Single antiplatelet	279 (26.4)	61 (30.7)	218 (25.5)	0.13
Dual antiplatelet	208 (19.7)	31 (15.6)	177 (20.7)	0.1
Oral anticoagulant	256 (24.3)	48 (24.1)	208 (24.3)	0.95
Anticoagulant + antiplatelet	111 (10.5)	18 (9)	93 (10.9)	0.45

Renal replacement therapy (*N*, %)	19 (1.8)	3 (1.5)	16 (1.9)	0.73
BMI (mean ± SD)	26 ± 4.8	26.6 ± 6.2	25.9 ± 4.7	0.06
EuroSCORE1 (mean ± SD)	14.2 ± 9.9	14.6 ± 10.9	14.1 ± 9.7	0.51
STS-PROM (mean ± SD)	6 ± 5.5	6.1 ± 5	5.9 ± 5.5	0.78

^∗^TAVI: transcatheter aortic valve implantation; CVRF: cardiovascular risk factors; NYHA: New York Heart Association; PCI: percutaneous coronary intervention; CABG: coronary artery bypass graft; BMI: body mass index.

**Table 2:** Characteristics of transcatheter aortic valve implantation (TAVI) procedures.

	Whole population (*N* = 1055)	Post-TAVI vascular complication group (*N* = 199)	Post-TAVI without vascular complication group (*N* = 856)	*p* value
TAVI indication (*N*, %)				<0.001
Severe aortic stenosis	986 (93.5)	180 (90.5)	806 (94.2)	
Others (aortic regurgitation/prosthetic valve degeneration)	69 (6.6)	19 (9.5)	50 (5.8)	

Right access site (*N*, %)	949 (90)	171 (85.9)	778 (90.9)	0.03

Introducer sheath size (*N*, %)				
14 Fr	632 (59.9)	101 (50.8)	531 (62)	0.04
16 Fr	329 (31.2)	75 (37.7)	254 (29.7)	
18 Fr	84 (8)	20 (10.1)	64 (7.5)	
20 Fr	10 (0.9)	3 (1.5)	7 (0.8)	

Valve types (*N*, %)				0.17
Edwards SAPIEN	538 (51)	96 (48.2)	442 (51.6)	
CoreValve Evolut	466 (44.2)	98 (49.2)	368 (43)	
ACURATE	51 (4.8)	5 (2.5)	46 (5.4)	

Valve size (*N*, %)				0.7
≤25	277 (26.3)	50 (25.1)	227 (26.5)	
]25–30]	687 (65.1)	129 (64.8)	558 (65.2)	
>30	91 (8.6)	20 (10.1)	71 (8.3)	

Procedure duration (*N*, %)				<0.001
≤60 min	154 (14.6)	22 (11.1)	132 (15.4)	
]60–120] min	864 (81.9)	159 (79.9)	705 (82.4)	
>120 min	37 (3.5)	18 (9)	19 (2.2)	

**Table 3:** Description of the observed vascular complications.

Type of vascular complications	*N* = 199 (%)
Aortic dissection	0.5
Aortic rupture	1.5
Unplanned endovascular stenting	15.6
Unplanned surgery	1.5
Ipsilateral lower extremity ischemia	0.5
Access site hematoma	69.8
Pseudoaneurysm	6
Arteriovenous fistula	1.5
Dissection	2
Residual nonsignificant stenosis	1

**Table 4:** Stepwise logistic regression studying the association between vascular complications, predictors, and adverse outcomes.

Variables	OR	95% CI	*p* value
Smoking	1.67	[0.58; 4.77]	0.33
Hypertension	1.42	[0.97; 2.08]	0.06
NYHA class	0.78	[0.6; 1.02]	0.07
TAVI indication	0.54	[0.29; 0.99]	0.04
Prior valvuloplasty	1.29	[0.79; 2.1]	0.3
Carotid disease	1.57	[0.71; 3.45]	0.26
BMI	1.02	[0.98; 1.05]	0.3
SAPT	1.17	[0.8; 1.7]	0.4
DAPT	0.73	[0.46; 1.16]	0.18
Right access site	0.6	[0.37; 0.99]	0.04
Introducer sheath size 14 Fr	0.63	[0.45; 0.89]	0.009
Valve types	0.82	[0.6; 1.1]	0.22
Procedure duration	1.8	[1.2; 2.8]	0.005
Death	1.63	[0.65; 4.09]	0.29
Bleeding	2.57	[1.8; 3.6]	<0.001
CCU stay (>24 hours)	2	[1.4; 3]	<0.001

^∗^NYHA: New York Heart Association; BMI: body mass index; SAPT: single antiplatelet therapy; DAPT: dual antiplatelet therapy; TAVI: transcatheter aortic valve implantation; CCU: cardiac care unit.

## Data Availability

The data used to support the findings of this study are available from the corresponding author upon request.
